# HLA-B*15:02 genotype associated with hypersensitivity syndrome to lamotrigine in Thai population

**DOI:** 10.1186/2045-7022-4-S3-P121

**Published:** 2014-07-18

**Authors:** Napatrupron Koomdee, Thawinee Jantararoungtong, Ticha Rerkpattanapipat, Santirat Prommas, Siwalee Santon, Montri Chammanphol, Apichaya Puangpetch, Chonlaphat Sukasem

**Affiliations:** 1 Ramathibodi Hospital, Laboratory for Pharmacogenomics, Thailand; 2 Ramathibodi Hospital, Medicine Department, Thailand

## Background

Lamotrigine is a commonly used in psychiatric patients. The aims of this study were to determine the possible associations of lamotrigine-induced hypersensitivity syndrome with the HLA-B alleles in Thai patients. HLA-B*58:01 allele was also observed in Han Chinese patients with epilepsy and lamotrigine-induced SCARs (severe cutaneous adverse drug reactions).

## Method

A total of 133 patients, including 11 patients with lamotrigine-induced hypersensitivity syndrome (MPE; maculo-papular exanthema, SJS; Stevens-Johnson syndrome and TEN; toxic epidermal necrolysis), 9 lamotrigine-tolerant controls and 113 healthy controls were included in this study. HLA-B genotyping was performed. This case-control study was approved by the Ethics Committee of Ramathibodi Hospital.

## Results

HLA-B*15:02 allele was present in 27.3% (3/11) of Thai patients with lamotrigine-induced hypersensitivity syndrome but in only 11.1% (1/9) of lamotrigine-tolerant controls and 13.2% of 113 healthy controls (P-value <0.05). Other HLA-B allele, including HLA-B*58:01 was observed in a case group.

## Conclusion

Our results support the hypothesis that HLA-B*15:02 contribute to the genetic susceptibility to lamotrigine-induced hypersensitivity syndrome and may be valuable as potential biomarkers for prediction lamotrigine-induced hypersensitivity syndrome in Thai population. A major limitation of this study was the size of the sample available for the analysis. Confirmation of these results in a larger, independent sample is needed.

